# Ethical principles and placebo-controlled trials – interpretation and implementation of the Declaration of Helsinki’s placebo paragraph in medical research

**DOI:** 10.1186/s12910-018-0262-9

**Published:** 2018-03-15

**Authors:** Antonia-Sophie Skierka, Karin B. Michels

**Affiliations:** 1 Faculty of Medicine and Medical Center, University of Freiburg, Freiburg, Germany; 2 Institute for Prevention and Cancer Epidemiology, Faculty of Medicine and Medical Center, University of Freiburg, Freiburg, Germany; 3 Department of Obstetrics, Gynecology and Reproductive Biology, Obstetrics and Gynecology Epidemiology Center, Brigham and Women’s Hospital, Harvard Medical School, Boston, MA USA; 4 Department of Epidemiology, Harvard School of Public Health, Boston, MA USA

**Keywords:** Placebo, Declaration of Helsinki, World medical association

## Abstract

**Background:**

In October 2013, the Declaration of Helsinki was revised a seventh time in its 50 year history. While it is the most widely accepted set of ethical principles for the protection of patients participating in medical research, the Declaration of Helsinki has also been subject of constant controversy. In particular, its paragraph on the use of placebo controls in clinical trials divides the research community into active-control and placebo orthodox proponents, both continuously demanding revisions of the Declaration of Helsinki in favour of their position. The goal of the present project is to compare the mainly theoretical controversy with regulatory implementation.

**Methods:**

We distributed a questionnaire to national drug regulatory authorities from different countries to collect information on the authorities’ respective approaches to interpretation and implementation of the Declarations’ placebo paragraph in the conduct of medical research.

**Results:**

Our findings suggest that the majority of drug regulatory authorities have established a practice of a middle ground, allowing placebo controls in some instances. Various interpretations of “serious harm” and “methodological reasons” are proposed as well as safeguards to avoid abuse of the option to use placebo-controls.

**Conclusion:**

Leaving the placebo paragraph open to various interpretation is a result of the Declaration of Helsinki’s character as a guidance document. With the current version controversy will continue. The Declaration should be continued to be strengthened to enforce the appreciation of conducting medical research with the highest ethical standard.

**Electronic supplementary material:**

The online version of this article (10.1186/s12910-018-0262-9) contains supplementary material, which is available to authorized users.

## Background

The Declaration of Helsinki emerged from the Nurnberg Code and provides the “Ethical Principles for Medical Research Involving Human Subjects”. Since 1964, when the Declaration of Helsinki was adopted by the World Medical Association, it represents one of the most respected set of ethical principles to guide medical researchers protecting patients enrolling in biomedical experimentation [1]. In October 2013, the Declaration of Helsinki underwent its seventh revision since its inception [2]. Despite its widely accepted authority, the Declaration of Helsinki has always been a document of constant change and controversy. One of the most contested paragraphs derives guidelines for the use of placebo in clinical trials.^1^ Over the years, two principal camps have formed that either defend placebo-controlled trials or advocate active-control trials. Representatives of both sides have called for changes of the placebo paragraph in favour of their stance by providing ethical and methodologic arguments in support of their respective positions.

The “placebo orthodox” camp considers placebo-controlled trials methodologically superior to active-control trials [3]. They provide mainly methodologic reasons to support their claim of ethicality of placebo-controlled trials even if effective treatment exists. According to them, placebo-controlled trials are necessary to distinguish between a treatment being efficacious and non-efficacious (assay sensitivity^2^) which is required to avoid ineffective drugs to be approved [4]. An additional argument, made in favour of placebo controls is the higher efficiency of placebo-controlled trials, as smaller study sizes are needed, fewer subjects are exposed to the uncertainties of the trials, which also makes drug development less costly [5]. Utilitarian arguments- justifying a few suffering for the good of an infinitive number of patients in the future are also made. [6] Some authors quantify the discomfort patients endure while participating in a PCT from tolerable and therefore ethically justifiable discomfort and non-justifiable risk of serious harm such as death or irreversible morbidity [4].

Contrary to “placebo orthodox”, “active-control orthodox” proponents do not accept arguments in favour of placebo-controlled trials in cases if proven effective treatment for the respective condition exists. Hence, the main premise of the Declaration of Helsinki, to protect the patient enrolling in biomedical research, can be maintained only by solely allowing active-control trials when proven effective treatment exists [3]. Firstly, placebo opponents see no justification of exposing the trial subjects to any kind of additional risk, harm or discomfort which could be prevented by providing active treatment [7]. Secondly, placebo-controlled trials breach the principle of equipoise, as placebo is inferior to standard treatment. Moreover, active-controlled trials are not only in concordance with equipoise but additionally allow a comparison of the treatment under investigation being superior, inferior or equal to standard treatment [8, 9]. Finally, defenders of active controls juxtapose the utilitarian argument by underlining the importance of the deontological principle which states that the obligation of the physician to protect his patients outweighs the gain of information for society [8].

The debate about the ethics of placebo-control is ever present. It has affected discussions on the testing of AIDS medications in developing countries [10, 11] as well as withholding active medication in patients with schizophrenia or depression in favour of placebo-controlled trials [12–15] and many other applications. The 2000 version of the Declaration of Helsinki’s placebo paragraph (Table 1) pursued a clear stance by only allowing placebo-controlled trials in cases in which no proven effective treatment existed. Due to several dismissive reactions to this unambiguous wording of the paragraph, a Note of Clarification was added in 2002 (Table 1), and the exceptions allowing placebo-controlled trials were expanded. The most recent version of the placebo paragraph states:

**Table 1:** Development of the paragraph on placebo use in the Declaration of Helsinki 1975–2013

Version of the Declaration of Helsinki	Paragraph concerning the use of placebo
29th WMA General Assembly, Tokyo, Japan, October 1975	“II.2 The potential benefits, hazards and discomfort of a new method should be weighed against the advantages of the best current diagnostic and therapeutic methods. II.3 In any medical study, every patient – including those of a control group, if any – should be assured of the best proven diagnostic and therapeutic method.” [23]
48th WMA General Assembly, Somerset West, South Africa, October 1996	“II.3 In any medical study, every patient – including those of a control group, if any – should be assured of the best proven diagnostic and therapeutic method. This does not exclude the use of inert placebo in studies where no proven diagnostic or therapeutic method exists”*.* [24]
52nd WMA General Assembly, Edinburgh, Scotland, October 2000	“§29 The benefits, risks, burdens and effectiveness of a new method should be tested against those of the best current prophylactic, diagnostic, and therapeutic methods. This does not exclude the use of placebo, or no treatment, in studies where no proven prophylactic, diagnostic or therapeutic method exists”*.* [25]
53rd WMA General Assembly, Washington DC, USA, October 2002	“§29 The benefits, risks, burdens and effectiveness of a new method should be tested against those of the best current prophylactic, diagnostic, and therapeutic methods. This does not exclude the use of placebo, or no treatment, in studies where no proven prophylactic, diagnostic or therapeutic method exists*.* Note of Clarification added: However, a placebo-controlled trial may be ethically acceptable, even if proven therapy is available, under the following circumstances: 1. Where for compelling and scientifically sound methodological reasons it is necessary to determine the efficacy or safety of a prophylactic, diagnostic or therapeutic method; or 2. Where a prophylactic, diagnostic or therapeutic method is being investigated for a minor condition and the patients who receive placebo will not be subject to any additional risk of serious or irreversible harm.” [26]
59th WMA General Assembly, Seoul, Korea, October 2008	“§32 The benefits, risks, burdens and effectiveness of a new intervention must be tested against those of the best current proven intervention, except in the following circumstances: -The use of placebo, or no treatment, is acceptable in studies where no current proven intervention exists; or -Where for compelling and scientifically sound methodological reasons the use of placebo is necessary to determine the efficacy or safety of an intervention and the patients who receive placebo or no treatment will not be subject to any risk of serious or irreversible harm. Extreme care must be taken to avoid abuse of this option.” [27]
64th WMA General Assembly, Fortaleza, Brazil 2013	“§33 The benefits, risks, burdens and effectiveness of a new intervention must be tested against those of the best proven intervention(s), except in the following circumstances: - Where no proven intervention exists, the use of placebo, or no intervention, is acceptable; or - Where, for compelling and scientifically sound methodological reasons, the use of any intervention less effective than the best proven one, the use of placebo, or no intervention is necessary to determine the efficacy or safety of an intervention and the patients who receive any intervention less effective than the best proven one, placebo, or no intervention will not be subject to additional risks of serious or irreversible harm as a result of not receiving the best proven intervention. Extreme care must be taken to avoid abuse of this option.” [2]

“§33: The benefits, risks, burdens and effectiveness of a new intervention must be tested against those of the best proven intervention(s), except in the following circumstances:Where no proven intervention exists, the use of placebo, or no intervention, is acceptable; or.Where, for compelling and scientifically sound methodological reasons, the use of any intervention less effective than the best proven one, the use of placebo, or no intervention is necessary to determine the efficacy or safety of an intervention.and the patients who receive any intervention less effective than the best proven one, placebo, or no intervention will not be subject to additional risks of serious or irreversible harm as a result of not receiving the best proven intervention.Extreme care must be taken to avoid abuse of this option” [2].

This latest revision does not differ much from the previous 2008 (Table 1) version. It still represents an attempt of a mid-way solution between active-control and placebo-orthodox positions: PCTs are allowed in cases, in which no standard treatment exists, or if compelling and scientifically sound methodolgic reasons are provided. Moreover, the patient should never be subject to an additional risk of serious or irreversible harm if withheld from standard treatment. Paragraph 33 adds that “any intervention less effective than the best proven one” is a justifiable alternative to the best proven treatment [2]. Hence, placebo, no treatment and, since the latest revision, every control inferior to the best proven treatment, such as second or third best standard treatment, is now justifiable under the above mentioned circumstances [16]. Additionally, the word “current” was erased, leaving, “Where no [current] proven intervention exists, the use of placebo, or no intervention, is acceptable.” Moreover, the new version clarifies that not “any” but “additional risks of serious or irreversible harm” must be avoided if denying the patient, the best proven treatment [2]. Nevertheless, the most recent version of the placebo paragraph does not differ much from the previous 2008 version, with respect to its position on placebo use in clinical trials. Placebo controls can still be justified under certain circumstances, even if active controls are favourable. However, the Declaration does not clarify situations in which placebo controls are acceptable and therefore offers room for interpretation.

While the question of whether and how to change the Declaration of Helsinki has been widely discussed on a theoretical level, an evaluation of its actual acceptance, interpretation, and implementation by drug regulatory authorities around the world is still lacking. We aimed to address this gap by setting out to answer the question of how the ethical principle on placebo use as laid down in the Declaration of Helsinki, is implemented in practice by national regulatory authorities in different countries. In order to provide an empirically informed answer to this question, we conducted a survey with national drug regulatory authorities around the world. We derived questions on the basis of ongoing theoretical discussions surrounding the ethical use of placebo controls in clinical trials. Our questionnaire referred to the 2008 version of the Declaration of Helsinki. As outlined above, the changes to the most recent version concerning the stance on the use of placebo in clinical trials are minor. Therefore, our findings are still applicable to the 2013 version.

### Implementation in medical research- a questionnaire to national regulatory authorities from different

To explore how the placebo stipulation of the Declaration of Helsinki is translated into medical research practice we conducted a survey of drug regulatory authorities around the world. We developed a questionnaire which we distributed to national drug regulatory authorities in 93 countries (See Additional file 1). With this survey, we aimed to evaluate the acceptance of the Declaration in practice, and to examine the relevance of other international guidelines. In a second step, we asked for the different interpretations and implementation of the Declaration of Helsinki’s placebo paragraph.

## Methods

Our study was reviewed by the Ethics Committee of the University of Freiburg, Germany, and considered not human research. Our aim was to include as many drug regulatory authorities as possible worldwide. We obtained the current official web addresses of the authorities from a “List of Globally identified Websites of Medicines Regulatory Authorities” of the WHO published in November 2012, providing the name of the country and the matching homepage of the authority (http://www.who.int/medicines/areas/quality_safety/regulation_legislation/en/).

We then accessed every home page to retrieve relevant contact information about the responsible head of the department for clinical trials of the respective regulatory authority. We chose to send the questionnaire via e-mail as it seemed to be the most convenient way to communicate with the regulatory authorities, especially in distant regions, as the questionnaire would reach them immediately and the authorities would not need to spend additional time and money for postal charges in order to reply. E-mail would also allow easy contact in case of any further questions. Moreover we received immediate response in case the e-mail delivery failed. We first designed the questionnaire using the questionnaire program “GrafStat” to allow the receiver to get to an online version following a link in our e-mail. As several firewalls blocked the link we additionally provided Word- and PDF-versions of the questionnaire. Initially, we only provided the questionnaire in English. Upon feedback from Latin American countries to provide a Spanish version, we translated the questionnaire into Spanish.

We sent out the first round of e-mails on the 27th of November in 2012, a follow-up to non-responders on the 13th of December 2012 and a third follow-up on the 14th of January 2013. If we did not receive a response to our questionnaire until February 2013, we tried to reach the authorities via telephone to verify the receipt of our questionnaire or to obtain further contact information. We sent a monthly reminder e-mail followed by phone calls every two months to those drug regulatory authorities we were able to contact, but which had not yet responded to our questionnaire until July 2013. For a few drug regulatory authorities we were unable to find relevant contact information mostly because of outdated e-mail and phone contact information by July 2013. We closed our survey in March 2014.

### The questionnaire

The Questionnaire (Additional file 1) consists of ten questions. The first two questions specify country and institution. The following eight questions pertain to the Declaration of Helsinki, in particular to the placebo paragraph. As we started our survey in November 2012, the questionnaire refers to the 2008 version of the Declaration of Helsinki, which was the most recent one at the time. We realize that the Declaration was revised in 2013 but since changes of the placebo paragraph were minor compared to 2008 our questionnaire is still fully applicable as discussed above. It therefore should not inflict the interpretation of our questionnaire. Four of the eight questions pertaining to the Declaration of Helsinki are multiple choice questions with the option of multiple answers; the other four questions are open ended. We communicated to the participants that we encouraged any additional comments on our questions which could be provided either in question ten or added to the respective multiple choice questions. The following sections present and discuss the results of the questionnaire.

## Results

In total we tried to contact 103 drug regulatory authorities. We succeeded to forward our questionnaire to 93 drug regulatory authorities worldwide (Additional file 2). We were not able to contact ten drug regulatory authorities because of their e-mail addresses and telephone numbers being out of service. From the 93 authorities contacted we received 42 responses to our inquiry (response rate 45%), while 51 authorities did not react to our request (55% non-responders). Of the 42 agencies who responded, 32 (34,4%) completed the questionnaire (Additional file 2). Ten countries refused to complete the questionnaire (Additional file 2) mostly because they did not consider themselves to be the appropriate authority to respond to the questions posed. Additional file 2 provides a list of participating countries and the attribution of each country to the corresponding authority.

### Regulatory authorities which refused to answer

Ten regulatory authorities (Additional file 2) responded to our request but refused to answer our questionnaire. The main reason they provided was that they did not consider themselves the correct authority on the topic (China, Hong Kong, Singapore, Vietnam, Sweden and Switzerland) and Denmark, Lithuania and Portugal referred to their Ethic Committees. Denmark, however, specified that they do “approve clinical trials with placebos” and that their “legislation is based on EU Directive 2001/20/EC”. Switzerland referred to EMA and FDA guidelines and indicated that “the main aim of the Helsinki Declaration serves to protect patients participating in clinical trials […]; the purpose is NOT to guide drug development or regulate the review/approval of new medicines”.

Australia did not provide any specific reason.

### Responses to the specific question

In the following, we provide the responses to the specific questions.

Q3: “Is your institution bound by the Declaration of Helsinki or do you follow other ethical guidelines for medical research?”

30 out of the 32 regulatory authorities which responded to our questionnaire chose the option “The Declaration of Helsinki is relevant” (Table 2). Health Canada which responded with a written text specified that it “has not adopted the Declaration of Helsinki”. The USA are guided by ICH-guidelines, which are referring to the principles of the Declaration of Helsinki, but not to any specific version. Ten countries additionally chose the second option “guided by other ethical principles”.

**Table 2:** Answers to question 3

a) The Declaration of Helsinki is relevant	b) Guided by other ethical principle
1. Austria-AGES/BASG	1. Canada
2. Argentina	2. Czech Republic
3. Armenia	3. EMA
4. Botswana	4. Germany
5. Chile	5. Israel
6. Cuba	6. Japan
7. Czech Republic	7. Namibia
8. EMA	8. Senegal
9. Germany	9. Turkey
10. Ghana	10. USA
11. Hungary	
12. Ireland	
13. Israel	
14. Japan	
15. Kenya	
16. Latvia	
17. Malaysia	
18. Namibia	
19. Republic of Belarus	
20. Saudi Arabia	
21. Sengal	
22. Slovakia	
23. Taiwan	
24. Tanzania	
25. The Netherlands	
26. Turkey	
27. UAE	
28. Uganda	
29. United Kingdom	
30. Zimbabwe	

Canada, Namibia, Turkey, USA, Senegal and Zimbabwe mentioned the ICH-guidelines E6, “Good Clinical Practice”. Five countries named the “local laws and regulations” to have to be considered; Austria states its Austrian Medicinal Product Act, the EU refers to those of its member states, Germany to its “Arzneimittelgesetz” (AMG) and “Good Clinical Practice-V” (GCP-V) laws, Israel to its “local law and regulations” and Japan to ethical guidelines published by the Ministry of Health, Labor and Welfare. Directives of the European Parliament and of the Council were also named among alternative guidelines from the Czech Republic (Directive 2005/28/EC) and Austria and the EU (Directive 2001/20/EC). Zimbabwe also refers to the guidelines of the Council for International Organizations of Medical Sciences (CIOMS).

Q4: “For the approval of a new pharmaceutical drug, do you require placebo controls or standard therapy for comparison in situations where effective treatment is available?”

No authority requires unconditional placebo controls for drug approval (Table 3). Twenty authorities require placebo controls in some instances. For nine authorities, those of Argentina, Armenia, Botswana, the Czech Republic, Hungary, Israel, Republic of Belarus, Slovakia and the United Kingdom, placebo controls are acceptable in the case of the disease not being life threatening. Ten regulatory authorities chose the option to use placebo controls in clinical trials “if the only burden on the patient is transient discomfort” such as, Botswana, Chile (“only if the design considers rescue medications and other measures”), the Czech Republic, the EU,^3^ Ireland, Israel, Latvia, Taiwan, The Netherlands and Germany (which specifies that placebo use is acceptable in the case “if no proven intervention is available”). Canada, Saudi Arabia and Turkey added “other” options, which mainly specify that the decision in the context of the Declaration of Helsinki is taken case-by-case. We counted these countries among those allowing placebo controls, as the Declaration justifies placebo controls in certain cases as discussed above. Nine regulatory authorities require exclusively standard therapy for a control group, namely Cuba, Ghana, Kenya, Malaysia, Namibia, Senegal, Tanzania, Uganda, and Zimbabwe among which are mainly (seven) African countries. The Czech Republic allows placebo control as well as standard therapy. The United Arab Emirates have similar provisions; moreover they recognize approval by authorities such as the FDA, MHRA, EMA and TGA. Japan requires standard therapy but evidence for new drug applications already approved by the FDA using placebo is considered if it is consistent with the Declaration of Helsinki. The USA specified that they allow several control choices and their “choice depends on what is appropriate”. They refer to the ICH E-10 guidelines which discuss among others placebo controls, non-inferiority trials as well as historical controls. Austria chose the option “other” without specifying.

**Table 3:** Answers to question 4

a) Always placebo control	b) Placebo control only if disease is not life threatening	c) Placebo control if the only burden on the patient is transient discomfort	d) Always standard therapy	e) Other
	1. Argentina	1. Botswana	1. Cuba	1. Austria
	2. Armenia	2. Chile	2. Czech Republic	2. Argentina
	3. Botswana	3. Czech Republic	3. Ghana	3.Germany
	4. Czech Republic	4. EMA	4. Japan	4. Japan
	5. Hungary	5. Germany	5 .Kenya	5. Saudi Arabia
	6. Israel	6. Ireland	6. Malaysia	6. Turkey
	7. Republic of Belarus	7. Israel	7. Namibia	7. UAE
	8. Slovakia	8. Latvia	8. Senegal	8. USA
	9. United Kingdom	9. Taiwan	9. Tanzania	
		10. The Netherlands	10. UAE	
			11. Uganda	
			12. Zimbabwe	

Q5: “The Declaration of Helsinki has been revised several times since 1964. Does your organization adhere to a specific version of the Declaration of Helsinki?”

Most of the regulatory authorities, twenty-two, are referring to the latest available version of the Declaration of Helsinki, including: Argentina, Armenia, Botswana, Chile, Cuba, EU, Germany, Ghana, Hungary, Ireland, Israel, Japan, Latvia, Malaysia, Republic of Belarus, Saudi Arabia, Senegal, Slovakia, The Netherlands, Turkey, United Arab Emirates and United Kingdom (Table 4). Fifteen of the countries following the latest version of the Declaration require placebo-controls in some instances (see question 4), namely Argentina, Armenia, Botswana, Chile, EU, Germany, Hungary, Ireland, Israel, Latvia, Republic of Belarus, Slovakia, The Netherlands, Turkey and United Kingdom. Solely three countries, Cuba, Ghana and Malaysia adhere to the latest version but require always standard therapy (see question 4). For Japan, Senegal and United Arab Emirates the version they adhere to is not clearly assignable to their research practice (question 4) due to multiple answers. Tanzania is referring to the 1975, 1996, 2000 and 2002 versions. The Czech Republic as well as Germany consider the 1996 version, whereas Germany specifies that “in Article 3 of the European Commission Directive 2005/28/EC the 1996 version of the Declaration is referred to” (Additional file 3). It should be noted that the Czech Republic stated in question 3 to additionally follow the Directive 2005/28/EC. Namibia and Zimbabwe adhere to the 2000 version and Uganda follows the 2002 version. As in question 4, the countries which appear to reject the most recent version are mainly African countries. Namibia and Zimbabwe as two of the three countries exclusively adhering to a version of the Declaration before 2002 state to always require placebo-controls (see question 4). Austria does not adhere to any specific version, as they are not bound by the Declaration of Helsinki. As the USA do not refer to the Declaration in any of its regulations, it does not adhere to any specific version either. Nevertheless, they commented that the Declaration “was reasonably ok on the matter of placebos until the 2000 version essentially banned placebo controls when there was any existing therapy […].This was largely repaired in the 2008 version.”

**Table 4:** Answers to question 5

a) 29th WMA General Assembly, Tokyo, Japan, October 1975	b) 48th WMA General Assembly, Somerset West, South Africa, October 1996	c) 52nd WMA General Assembly, Edinburgh, Scotland, October 2000	d) 53rd WMA General Assembly, Washington DC, USA, October 2002	e) 59th WMA General Assembly, Seoul, Korea, October 2008	f) Other
1. Senegal	1. Czech Republic	1. Namibia	1. Tanzania	1. Armenia	1. Austria
2. Tanzania	2. Senegal	2. Senegal	2. Uganda	2. Botswana	2. Argentina
	3.Tanzania	3. Tanzania	3. Senegal	3. Chile	3. Germany
	4. Germany	4. Zimbabwe		4. Cuba	4. Japan
				5. EMA	5. USA
				6. Ghana	
				7. Hungary	
				8. Ireland	
				9. Israel	
				10. Latvia	
				11. Malaysia	
				12. Republic of Belarus	
				13. Saudi Arabia	
				14. Senegal	
				15. Slovakia	
				16. The Netherlands	
				17. Turkey	
				18. UAE	
				19. United Kindom	

Q6: “How do you interpret paragraph 32 of the Declaration of Helsinki?”

No one interprets the paragraph on placebo use as placebo controls being appropriate in every situation (Table 5). Twenty-three of the regulatory authorities, namely Argentina, Armenia, Botswana, Chile, Czech Republic, EU, Ghana, Hungary, Ireland, Israel, Kenya, Latvia, Malaysia, Namibia, Republic of Belarus, Saudi Arabia, Senegal, Slovakia, Taiwan, Tanzania, The Netherlands, United Arab Emirates and Zimbabwe, understand the wording of the paragraph as a suggestion to avoid placebo control whenever possible if effective treatment is available. Fourteen of these authorities state in question 4 that they require placebo controls in some instances, namely Argentina, Armenia, Botswana, Chile, EU, Hungary, Ireland, Israel, Latvia, Republic of Belarus, Saudi Arabia, Slovakia, Taiwan and The Netherlands. Seven countries, Ghana, Kenya, Malaysia, Namibia, Senegal, Tanzania and Zimbabwe always demand standard therapy in medical research (see question 4). Four authorities, those of Israel, Senegal, Uganda and United Kingdom, read the paragraph to allow placebo controls if “effective treatment exists but is not available in the location where the study is conducted.” Eight authorities chose the option “other” to explain their interpretation of the placebo paragraph. Austria referred to its ethic committee for any ethical aspects on clinical trials. Cuba, Japan, Turkey, USA and Germany understand that the paragraph does not necessarily confirm placebos unethical when proven effective treatment exists. Turkey specifies that their interpretation depends on the disease and the effective treatment whereas Germany refers to the “compelling and scientifically sound methodological reasons” [2] which could justify a placebo control under the condition that “the duration of the placebo treatment is as short as necessary”. The USA emphasizes that in most symptomatic conditions a placebo is needed to interpret results. Additionally they specify that denial of available treatment is only acceptable if resulting in discomfort, not in harm, and if the patients are fully informed and not coerced.

**Table 5:** Answers to question 6

a) The use of placebo controls is appropriate in any circumstance	b) The use of placebo control should be avoided whenever possible if effective treatment is available	c) The use of placebo is appropriate even if effective treatment exists but is not available in the location where the study is conducted	d) Other
	1. Argentina	1. Israel	1. Austria
	2. Armenia	2. Senegal	2. Cuba
	3. Botswana	3. Uganda	3. Germany
	4. Chile	4. United Kingdom	4. Japan
	5. Czech Republic		5. Saudi Arabia
	6. EMA		6. Turkey
	7. Ghana		7. USA
	8. Hungary		
	9.I reland		
	10. Israel		
	11. Kenya		
	12. Latvia		
	13. Malaysia		
	14. Namibia		
	15. Republic of Belarus		
	16. Saudi Arabia		
	17. Senegal		
	18. Slovakia		
	19. Taiwan		
	20. Tanzania		
	21. The Netherlands		
	22. UAE		
	23. Zimbabwe		

Q7 to Q9:

The answers to open-ended questions are provided in Tables 6, 7 and 8.

**Table 6:** Answers to question 7. “What are “compelling and scientifically sound methodological reasons” as outlined in paragraph 32 of the Declaration of Helsinki for your institution which would justify the use of placebo?”

Compelling and scientifically sound methodological reasons	Country
Questionable effectiveness of standard treatment	Armenia, Canada, Cuba, Israel, Namibia
Assay sensitivity/ when placebo is the most rigorous test of efficacy	Germany, Ghana, USA, The EU
No current proven intervention exists	Namibia, Uganda, Zimbabwe
High placebo response rate	Canada, Chile, Cuba
None if effective treatment exists	Japan, United Arab Emirates, Zimbabwe
Standard treatment is too toxic	Ghana, Tanzania
Available treatment is too expensive	Botswana, Uganda
Non-responders	Israel, Tanzania
Add on	Ghana, Saudi Arabia
Size of placebo groups may be smaller than in active control studies	Saudi Arabia

**Table 7:** Answers to question 8. “How does your institution define “serious harm” as outlined in paragraph 32 of the Declaration of Helsinki for a patient which would restrict the use of placebo?”

Definition of serious harm	Country
Persistent or significant disability/incapacity	Armenia, Botswana, Germany, Ghana, Japan, Latvia, Malaysia, Namibia, Republic of Belarus, Saudi Arabia, Slovakia, The EU, The Netherlands, USA
Life-threatening events	Botswana, Cuba, Czech Republic, Germany, Ghana, Hungary, Israel, Namibia, Republic of Belarus, Saudi Arabia, Tanzania, The EU
Death	Botswana, Ghana, Namibia, Republic of Belarus, Saudi Arabia, Tanzania, The EU, USA
Inpatient hospitalization or causes prolongation of existing hospitalization	Germany, Ghana, Namibia, Saudi Arabia, The EU, United Arab Emirates
Congenital anomaly/birth defect	Germany, Ghana, Namibia, Saudi Arabia,
Requires intervention to prevent permanent impairment or damage	Saudi Arabia, The EU
Maintenance therapy for schizophrenic patients	Hungary

**Table 8:** Answers to question 9. “Which measures does your institution take “to avoid abuse of this option” as outlined in paragraph 32 of the Declaration of Helsinki?”

Safeguards	Country
Ethic Committees/International Review Boards (IRBs)	Austria, Argentina, Cuba, Germany, Ireland, Malaysia, Namibia, Republic of Belarus, Senegal, The EU, United Arab Emirates, Uganda, USA
Trial protocol^a^	Canada, Czech Republic, Ghana, Israel, Slovakia, The EU, The Netherlands,
During the trial: closely monitoring, short time period of trial, rescue medication, patients right to withdraw from the trial at any time	Chile, Cuba, the EU, Republic of Belarus Saudi Arabia
Rejection of trial if not in accordance with requirements	Austria, Czech Republic, The EU, USA
No difference between local and global standard	Botswana, The EU
Add-on study	Germany
No placebo use if effective treatment is available	Japan
Informed consent	The EU
None	United Kingdom

^a^Including: a justification for placebo use, a comparison of placebo to standard therapy and an outline of the methodology that would be used to minimize the risk to trial subjects

## Discussion

Our findings suggest that the majority of regulatory drug authorities requires placebo controls in some instances for controlled drug trials and therefore implement a middle ground as suggested by the Declaration of Helsinki’s placebo paragraph, which neither fully rejects nor accepts placebo controls.

Secondly, regarding the implementation of the placebo paragraph by regulatory drug authorities, the Declaration of Helsinki as a guidance document, leaves room for various interpretations of “serious harm”, “compelling and scientifically sound methodological reasons” and how to avoid abuse of the option to use placebo controls. Therefore a universally accepted standard does not seem to be established.

Thirdly, when it comes to research ethics, the Declaration of Helsinki remains the most widely respected document and establishes a minimum standard for guidance in medical research.

We have surveyed drug regulatory authorities worldwide to elucidate interpretation and application of the Declaration of Helsinki when governing the testing of drug treatments in humans. This study is the first to extend the discussions on the placebo paragraph of the Declaration of Helsinki to a practical level by evaluating which trial design is internationally mandated in practice when conducting medical experiments. Our findings are important as they underscore the significance of the Declaration of Helsinki in international research practice but simultaneously unveil vagueness in the Declaration that permit considerable variation in interpretation and application.

### A middle ground in medical research

As outlined above, the Declaration of Helsinki attempts to compromise between active-control and placebo orthodoxy, without fully accommodating any of the two positions. Our questionnaire indicates that this middle ground appears to be implemented in practice by the regulatory authorities: effective treatment is favorable, but placebo controls can be justified in certain situations and with additional safeguards. The Declaration does not stipulate on how to interpret “serious harm” and “compelling scientifically sound methodological reasons” but allows the local regulators and independent review boards decide on that matter. The regulatory authorities’ answers to our questionnaire mirror most of the discussed variety of interpretation of the Declarations’ placebo paragraph.

Defining serious harm: Placebo as well as active control defenders agree that death or irreversible morbidity resulting from the denial of proven effective therapy present sufficient “serious harm” to not use a placebo control [4]. Further definitions of “serious harm” suggested by the regulatory authorities (Table 3) comply with the definition of “serious adverse event” as laid down in the GCP-guideline and include:

“Any untoward medical occurrence that at any dose: results in death, is life threatening, requires inpatient hospitalization or prolongation of existing hospitalization, results in persistent or significant disability/incapacity, or is a congenital anomaly/birth defect” [17].

A missing element in this definition is the psychological and social harm which may be caused by withholding effective treatment to the patient, which are equally important to consider [3].

Defining “methodological reasons”: The Declaration of Helsinki states that “compelling and scientifically sound methodological reasons” may justify the use of placebo in a clinical trial. As pointed out earlier, there are some reasons accepted from both camps for placebo use in clinical trials: if no current proven intervention for the respective condition exists, or if a patient population that is not responding to available treatment [8]. Our survey results suggest (Table 2) that the majority of regulatory authorities interpret the “methodological reasons” in the above mentioned way. Nevertheless, several responses also mirror various other methodologic justifications often quoted by defenders of placebo controls:

- Active controls’ lack of assay sensitivity.
- Standard treatment is not always effective.
- Smaller sample sizes are required for superiority trials.

Another methodologic justification for placebo use mentioned by two resource-poor countries, Botswana and Uganda, is that placebos may be considered if existing treatment is too expensive for the majority of the population in the respective country.

Defining safeguards: In situations in which there may not be risk of serious harm and there are strong methodologic reasons in favor of placebo controls (e.g. a non-responder population), safeguards are still necessary. Our questionnaire suggests (Table 4) that in practice safeguards such as review by IRBs, informed consent, trial protocols, regular monitoring of the patient, and rescue medication are used to protect the well-being of the patient when enrolling in a placebo-controlled trial Regulatory authorities propose various safeguards to protect the wellbeing of the patient in placebo-controlled trials. If a placebo-controlled trial has been found acceptable, regulatory authorities suggest that participants should be closely monitored, have the possibility to withdraw from the trial at any time, receive rescue medication in case their condition worsens and the trial length should be as short as possible. Therefore, the latter reasoning does not justify the use of placebo; these measures should be taken in a placebo-controlled trial (or any trial for that matter) independent of the ethical considerations. In this context, the UK regulatory authorities’ reply, stating no measures are taken to avoid abuse of this option, may be an outlier.

When dealing with the phrasing of the Declaration of Helsinki’s placebo paragraph, one should take into consideration the character of the document. The Declaration is a guidance document. Therefore, it represents the ethical considerations and the minimum ethical standard of the WMA member states when conduction human research. Hence, it could be argued, that it would be against the Declaration’s character to set up clear definitions of “serious harm” or “methodological reasons”, as it is not a rigid law but often described as a “living document” [18], being under continuing revisions and discussions concerning the current ethical issues when conduction human research. Room for interpretation can be understood as a source of potential ethical abuse or it can bear the chance for local regulators to set up regulations based on the Declarations principles adapted to their local and occasional issues. Nevertheless, the protection of the patient should remain the highest priority and a double standard in research ethics should be avoided.

### The declaration of Helsinki: A respected guideline

Our results suggest that the Declaration of Helsinki is a relevant document of significant international impact providing ethical guidance for medical research worldwide. Besides the Declaration of Helsinki, other ethical guidelines such as the CIOMS-guidelines as well as local laws, regulations and regulatory documents such as the ICH-guidelines and Directives of the EU also seem to be considered by regulatory authorities in the context of drug approval (Additional file 3). The ICH- regulations and CIOMS-guidelines both refer to the Declaration of Helsinki, underlining its international value and acceptance. The Declaration, a document published by the WMA represents the ethical considerations of 106 national medical associations, from both developed and developing countries and seems to be the most widely accepted ethical guideline [19]. The ICH-guidelines, which is a regulatory document and therefore legally binding are followed by six members including USA, Japan and several EU countries. They consider the option of placebo-controlled trials even if effective treatment is known to be life-saving or to prevent irreversible morbidity if certain designs are used such as: “add-on, replacement, early escape, brief placebo period, and randomized withdrawal”, ([20] E-10 p. 29). The CIOMS-guideline is mainly designated to resource-poor countries and discusses extensively in its “Commentary on Guideline 5” under which situations a placebo may be used as a comparator ([21], pp. 15–19). The guidelines also address the difficulty of conducting placebo-controlled trials in countries where the effective treatment is not available ([21], pp. 18–19).

### Is the declaration suitable to guide the use of placebo controls?

Some might argue, that our findings suggest that the Declaration of Helsinki is not suitable to guide drug development in practice. To counter this argument, it is necessary to clarify to whom the Declaration is addressed. In the second paragraph of the 2013 version, the Declaration states that it is “addressed primarily to physicians” but “encourages others who are involved in medical research involving human subjects to adopt these principles” [2]. This clearly states that not only physicians but also clinical investigators and International Review Boards (IRBs) should take the Declaration of Helsinki into consideration; and they do, as our questionnaire suggested.

### Is the sample of drug regulatory authorities representative?

We distributed the questionnaire to various Drug Regulatory Authorities in different regions around the world. Considering the World Bank atlas method (https://datahelpdesk.worldbank.org/knowledgebase/articles/906519-world-bank-country-and-lending-groups) to designate a country as “low-income economies” (LIE), “lower-middle-income economies” (LMIE), “upper-middle-income economies” (UMIE) and “high-income economies” (HIE) in relation to the GNI per capita, four LIE-, three LMIE-, seven UMIE- and seventeen HIE-countries completed the questionnaire. Drug Regulatory Authorities from East Asia and Pacific (Three countries), Europe and Central Asia (twelve countries), Latin America and the Caribbean (three countries), North America (two countries) and Sub Saharan Africa (eight countries) are represented by our study. South Asia is not represented.

### Limitations and weaknesses of the study

We are aware of the fact that our study has its limitations and weakness. Firstly, we weren’t able to receive answers from every of the contacted drug regulatory authorities. Therefore, our results represent a convenience sample of the international drug regulatory authorities’ research practice.

Secondly, in retrospective the option of multiple choice answers resulted in a more difficult interpretation and in some cases even led to uninterpretable results- demanding a definite, single choice answer would have been preferable.

Thirdly, the questionnaire was only distributed in two languages (English and Spanish). Even though no request translating the questionnaire to other languages (e.g. French) has been put forward to us, various language options might have increased the response rate.

Lastly, our study is not referring to the latest version of the Declaration of Helsinki, which was published after we initiated our survey, however, the results are applicable to the 2013 version.

## Conclusion

Even after fifty years of constant revisions the Declaration of Helsinki remains an ambivalent document concerning “ethical principles for medical research involving human subjects” [2]. We focused our study on the topic of placebo use in cases when proven effective treatment exists. With the goal of obtaining information on how the Declaration of Helsinki is accepted and its principle on placebo use is interpreted and implemented by regulatory authorities we conducted a study by distributing a questionnaire to national regulatory authorities in different countries. The results indicate that the Declaration of Helsinki is the most accepted and adopted ethical guideline on medical research involving human subjects and seen as a minimum ethical standard by international drug regulatory authorities. Even though other local and international statements are considered, none takes precedence over the ethical principles laid down in the Declaration. In concordance with the Declaration’s phrasing of the placebo paragraph, the majority of drug regulatory authorities are requiring placebo-controlled trials under certain circumstances. This middle ground is the result of the Declaration of Helsinki’s character as a guidance document, leaving “serious harm”, “methodological reasons” as well as safeguards to avoid abuse of placebo-controlled trials ambiguous and open to various interpretations. With the current version of the Declaration of Helsinki controversy will continue and revisions in favor of both camps will be claimed. It is important to protect the process of revision from pressure of stakeholders and therefore maintain the high ethical standards for international research with the Declaration’s aims to protect.

## Additional files

Additional file 1: Questionnaire. Contains the Questionnaire we developed and sent to the national drug regulatory authorities in different countries. (DOCX 21 kb)

Additional file 2: Participating countries and corresponding DRA. Contains a table listing the drug regulatory authorities replying to our request and which of those completed the questionnaire or refused to complete it. (DOCX 17 kb)

Additional file 3: Evaluation of the Questionnaire. Provides several tables including the original answers from all participating countries to each question of the questionnaire. (DOCX 194 kb)

## Abbreviations

CIOMS: Council for International Organizations of Medical Sciences
EMA: European Medicines Agency
FDA: Food and Drug Administration
GCP: Good clinical practice
GNI: Gross national income
HIE: High-income economies
ICH: Interntional Conference on Harmonisation
IRB: Institutional review board
LIE: Low-income economies
LMIE: Lower-middle-income economies
MHRA: Medicines and Healthcare Products Regulatory Agency
PCT: Placebo controlled trial
TGA: Therapeutic Goods Administration
The EU: The European Union
The UK: The United Kingdom
The USA: The United States of America
UMIE: Upper-middle-income economies
WHO: World Health organisation
WMA: World Medical Association

## Footnotes

1 In the most recent 2013 version of the Declaration of Helsinki, guidance on the use of placebo in clinical trials is outlined in paragraph 33 [2]. As the numbering of the paragraph has changed through the several revisions of the Declaration, we will refer to it as “placebo paragraph” in the following.

2 One of the methodological arguments in favor of placebo-controlled trials indicates that the assay sensitivity, the ability of a trial to distinguish between an efficacious and non-efficacious treatment, is better in placebo-controlled than in active-control trials, which are often non-inferiority trials [4]. The FDA fears that a lack of assay sensitivity in active-control studies would result in the approval of ineffective drugs ([22])

3 We are aware that the EU is not a country but a union of states. In the following, to avoid confusion and as the EU has its own drug regulatory authority, the European Medicines Agency (EMA), we will continue to list the EU among “countries”.
