# Supplementary material for: Ethical principles and placebo-controlled trials – interpretation and implementation of the Declaration of Helsinki’s placebo paragraph in medical research
Source: BMC Med Ethics. 2018 Mar 15;19:24. doi: 10.1186/s12910-018-0262-9 (PMC5856313; doi:10.1186/s12910-018-0262-9)
Supplement: Supplementary file 1 — Questionnaire. Contains the Questionnaire we developed and sent to the national drug regulatory authorities in different countries. (DOCX 21 kb) [file 12910_2018_262_MOESM1_ESM.docx]

**Supplement 1: Questionnaire**

1. Name of your country:

2. Name of your institution:

3. Is your institution bound by the Declaration of Helsinki or do you follow other ethical guidelines for medical research?

The Declaration of Helsinki is relevant

Guided by other ethical principle. Please specify:

4. For the approval of a new pharmaceutical drug, do you require placebo controls or standard therapy for comparison, in situations where effective treatment is available?

Always placebo control

Placebo control only if disease is not life threatening.

Placebo control if the only burden on the patient is transient discomfort.

Always standard therapy

Other:

5. The Declaration of Helsinki has been revised several times since 1964. Does your organization adhere to a specific version of the Declaration of Helsinki? *[If you wish to refer back to the paragraphs pertaining to the use of placebo controls please see the appendix]*

29th WMA General Assembly, Tokyo, Japan, October 1975

48^th^ WMA General Assembly, Somerset West, South Africa, October 1996

52^nd^ WMA General Assembly, Edinburgh, Scotland, October 2000

53^rd^ WMA General Assembly, Washington DC, USA, October 2002

59^th^ WMA General Assembly, Seoul, Korea, October 2008

Other:

6. The current version of the Declaration of Helsinki of 2008 specifies:

“The benefits, risks, burdens and effectiveness of a new intervention must be tested against those of the best current proven intervention, except in the following circumstances:

- The use of placebo, or no treatment, is acceptable in studies where no current proven intervention exists; or
- Where for compelling and scientifically sound methodological reasons the use of placebo is necessary to determine the efficacy or safety of an intervention and the patients who receive placebo or no treatment will not be subject to any risk of serious or irreversible harm. Extreme care must be taken to avoid abuse of this option.”^[[1]](#footnote-1)^

How do you interpret paragraph 32 of the Declaration of Helsinki?

The use of placebo controls is appropriate in any circumstance.

The use of placebo control should be avoided whenever possible if effective treatment is available.

The use of placebo is appropriate even if effective treatment exists but is not available in the location where the study is conducted.

Other:

7. What are “compelling and scientifically sound methodological reasons” as outlined in paragraph 32 of the Declaration of Helsinki (see above) for your institution which would justify the use of placebo?

8. How does your institution define “serious harm” as outlined in paragraph 32 of the Declaration of Helsinki (see above) for a patient which would restrict the use of placebo?

9. Which measures does your institution take “to avoid abuse of this option” as outlined in paragraph 32 of the Declaration of Helsinki (see above)?

10. Do you have any additional comments on any of our questions above?

*Thank you very much for you participation. Your help is much appreciated*!

| Version of the Declaration of Helsinki | Paragraph concerning the use of placebo |
| --- | --- |
| 29th WMA General Assembly, Tokyo, Japan, October 1975 | “II.2 The potential benefits, hazards and discomfort of a new method should be weighed against the advantages of the best current diagnostic and therapeutic methods.  II.3 In any medical study, every patient – including those of a control group, if any – should be assured of the best proven diagnostic and therapeutic method.”^[[2]](#footnote-2)^ |
| 48^th^ WMA General Assembly, Somerset West, South Africa, October 1996 | “II.3 In any medical study, every patient – including those of a control group, if any – should be assured of the best proven diagnostic and therapeutic method. This does not exclude the use of inert placebo in studies where no proven diagnostic or therapeutic method exists”*.^[[3]](#footnote-3)^* |
| 52^nd^ WMA General Assembly, Edinburgh, Scotland, October 2000 | “§29:The benefits, risks, burdens and effectiveness of a new method should be tested against those of the best current prophylactic, diagnostic, and therapeutic methods. This does not exclude the use of placebo, or no treatment, in studies where no proven prophylactic, diagnostic or therapeutic method exists”*.^[[4]](#footnote-4)^* |
| 53^rd^ WMA General Assembly, Washington DC, USA, October 2002 | “§29: The benefits, risks, burdens and effectiveness of a new method should be tested against those of the best current prophylactic, diagnostic, and therapeutic methods. This does not exclude the use of placebo, or no treatment, in studies where no proven prophylactic, diagnostic or therapeutic method exists*.*  Note of Clarification added:  1. Where for compelling and scientifically sound methodological reasons it is necessary to determine the efficacy or safety of a prophylactic, diagnostic or therapeutic method; or  2. Where a prophylactic, diagnostic or therapeutic method is being investigated for a minor condition and the patients who receive placebo will not be subject to any additional risk of serious or irreversible harm.”^[[5]](#footnote-5)^ |
| 59^th^ WMA General Assembly, Seoul, Korea, October 2008 | “§32: The benefits, risks, burdens and effectiveness of a new intervention must be tested against those of the best current proven intervention, except in the following circumstances:  -The use of placebo, or no treatment, is acceptable in studies where no current proven intervention exists; or  -Where for compelling and scientifically sound methodological reasons the use of placebo is necessary to determine the efficacy or safety of an intervention and the patients who receive placebo or no treatment will not be subject to any risk of serious or irreversible harm. Extreme care must be taken to avoid abuse of this option.”^[[6]](#footnote-6)^ |

Appendix

1. World Medical Association. Declaration of Helsinki. Amended by the 59^th^ WMA General Assembly, Seoul, Korea, October 2008 [↑](#footnote-ref-1)
2. World Medical Association. Declaration of Helsinki. Amended by the 29^th^ WMA General Assembly, Tokyo, Japan, October 1975 [↑](#footnote-ref-2)
3. World Medical Association. Declaration of Helsinki. Amended by the 48^th^ WMA General Assembly, Somerset West, South Africa, October 1996 [↑](#footnote-ref-3)
4. World Medical Association. Declaration of Helsinki. Amended by the 52^th^ WMA General Assembly, Edinburgh, Scotland, October 2000. [↑](#footnote-ref-4)
5. World Medical Association. Declaration of Helsinki. Amended by the 53^th^ WMA General Assembly, Washington, DC, USA, October 2002. [↑](#footnote-ref-5)
6. World Medical Association. Declaration of Helsinki. Amended by the 59^th^ WMA General Assembly, Seoul, Korea, October 2008 [↑](#footnote-ref-6)
