# Supplementary material for: Ethical principles and placebo-controlled trials – interpretation and implementation of the Declaration of Helsinki’s placebo paragraph in medical research
Source: BMC Med Ethics. 2018 Mar 15;19:24. doi: 10.1186/s12910-018-0262-9 (PMC5856313; doi:10.1186/s12910-018-0262-9)
Supplement: Supplementary file 2 — Participating countries and corresponding DRA. Contains a table listing the drug regulatory authorities replying to our request and which of those completed the questionnaire or refused to complete it. (DOCX 17 kb) [file 12910_2018_262_MOESM2_ESM.docx]

**Supplement 2: Participating countries and corresponding drug regulatory authority**

| Country | Drug regulatory authority |
| --- | --- |
| 1. ***Argentina*** | ***Administración National de Medicamentos, Alimentos y Technología Médico (ANMAT)*** |
| 1. ***Armenia*** | ***The Scientific Centre of Drug and Medical Technology Expertise*** |
| 1. Australia | Therapeutic Goods Administration (TGA) |
| 1. ***Austria*** | ***BASG- Federal Office for Safety in Health Care***  ***AGES- Austrian Agency for Health and Food Safety*** |
| 1. ***Botswana*** | ***Ministry of Health, Drugs Regulatory Unit*** |
| 1. ***Canada*** | ***Health Canada*** |
| 1. ***Chile*** | ***La Agencia Nacional de Medicamentos del Instituto de Salud Pública de Chile (ANAMED)*** |
| 1. China | State Food and Drug Administration |
| 1. ***Cuba*** | ***The National Coordinating Center of Clinical Trials (CENCEC)*** |
| 1. ***Czech Republic*** | ***State Institute for Drug Control*** |
| 1. Denmark | The Danish Health and Medicines Authority |
| 1. ***Germany*** | ***Bundesinstitut für Arzneimittel und Medizinprodukte (BfArM, Federal Institut for drugs and medical devices)*** |
| 1. ***Ghana*** | ***Food and Drugs Authority*** |
| 1. Hong Kong | Department of Health, Drug Office |
| 1. ***Hungary*** | ***The National Institute of Pharmacy*** |
| 1. ***Ireland*** | ***Irish Medicines Board*** |
| 1. ***Israel*** | ***Ministry of Health, Pharmaceutical Division*** |
| 1. ***Japan*** | ***Risk Analysis Research Center, The Institute of Statistical Mathematics^[[1]](#footnote-1)^*** |
| 1. ***Kenya*** | ***Pharmacy and poisons Board Kenya*** |
| 1. ***Latvia*** | ***State Agency of Medicines*** |
| 1. Lithuania | State Medicines Control Agency of Lithuania |
| 1. ***Malaysia*** | ***National Pharmaceutical Control Bureau*** |
| 1. ***Namibia*** | ***Namibia Medicines Regulatory Council*** |
| 1. Portugal | INFARMED - National Authority of Medicines and Health Products, I.P. |
| 1. ***Republic of Belarus*** | ***Ministry of Health – Center for Examinations and Tests in Health Services*** |
| 1. ***Saudi Arabia*** | ***Saudi Food and Drug Authority*** |
| 1. ***Senegal*** | ***Direction de la pharmacie et du medicament*** |
| 1. Singapore | Health Science Authority |
| 1. ***Slovakia*** | ***State Institute for Drug Control*** |
| 1. Sweden | Medical Products Agency (MPA) |
| 1. Switzerland | Swissmedic, Schweizerisches Heilmittelinstitut |
| 1. ***Taiwan*** | ***Food and Drug Administration*** |
| 1. ***Tanzania*** | ***Tanzania Food and Drugs Authority (TFDA)*** |
| 1. ***The European Union*** | ***European Medicines Agency (EMA)*** |
| 1. ***The Netherlands*** | ***Medicines Evaluation Board;***  ***Central Committee on Research Involving Human Subjects*** |
| 1. ***Turkey*** | ***Ministry of Health, Directorate of Pharmacy and Pharmaceuticals*** |
| 1. ***Uganda*** | ***National Drug Authority, Uganda*** |
| 1. ***United Arab Emirates*** | ***Registration and Drug Control Department, Ministry of Health*** |
| 1. ***United Kingdom*** | ***Medicines and Healthcare Products Regulatory Agency (MHRA)*** |
| 1. ***USA*** | ***Food and Drug Administration (FDA)*** |
| 1. Vietnam | Drug Administration of Vietnam |
| 1. ***Zimbabwe*** | ***Medicines Control Authority of Zimbabwe*** |

***Drug Regulatory Authorities that completed our questionnaire***

Drug Regulatory Authorities that refused to answer our questionnaire

1. The drug regulatory authority of Japan, the PMDA, refused to answer our questionnaire. A former PMDA advisory committee member completed our questionnaire from a personal view point of PMDA policy. [↑](#footnote-ref-1)
