# Supplementary material for: Ethical principles and placebo-controlled trials – interpretation and implementation of the Declaration of Helsinki’s placebo paragraph in medical research
Source: BMC Med Ethics. 2018 Mar 15;19:24. doi: 10.1186/s12910-018-0262-9 (PMC5856313; doi:10.1186/s12910-018-0262-9)
Supplement: Supplementary file 3 — Evaluation of the Questionnaire. Provides several tables including the original answers from all participating countries to each question of the questionnaire. (DOCX 194 kb) [file 12910_2018_262_MOESM3_ESM.docx]

**Supplement 3: Evaluation of the questionnaire**

| Question 3: Is your institution bound by the Declaration of Helsinki or do you follow other ethical guidelines for medical research? |  |
| --- | --- |
| a) The Declaration of Helsinki is relevant | b) Guided by other ethical principle |
| 1. Austria: The Declaration of Helsinki is not a legally binding document, so BASG/AGES cannot be "bound" by it. However, international and national legislation refer to the principles as laid down in the Declaration of Helsinki, e.g. Directive  2001/20/EC or the Austrian Medicinal Product Act. | 1. Canada: Please be advised that Health Canada has not adopted the Declaration of  Helsinki. Rather,we refer clinical trial sponsors to the Regulations that govern clinical research (Drugs For Clinical Trials Involving Human Subjects), as well as the internationally-recognized guidelines developed by the International Conference on Harmonisation (ICH) - Good Clinical Practices E6(R1) (May 1996). ICH E6 is the accepted standard for the design, conduct, recording and reporting of drug clinical trials that involve the participation of human subjects. |
| 2. Argentina | 2.Czech Republic : Directiva 2005/28/EC |
| 3. Armenia | 3.EMA: According to the paragraph 8 of the preamble – Introduction and General Principles of Annex 1 to Directive 2001/83/EC all clinical trials, conducted within the European Community, must comply with the requirements of Directive 2001/20/EC of the European Parliament and of the Council on the approximation of the laws, regulations  and administrative provisions of the Member States relating to the implementation of  good clinical practice in the conduct of clinical trials on medicinal products for human use. To be taken into account during the assessment of an application, clinical trials,  conducted outside the European Community, which relate to medicinal products intended to be used in the European Community, shall be designed, implemented and reported on what good clinical practice and ethical principles are concerned, on the basis of  principles, which are equivalent to the provisions of Directive 2001/20/EC. They shall be carried out in accordance with the ethical principles that are reflected, for example, in the Declaration of Helsinki. |
| 4. Botswana | 4.Germany: Applicable ethical principles in the national law (AMG, GCP-V) |
| 5. Chile | 5. Israel: Our local law and regulations. |
| 6. Cuba | 6.Japan: Ethical guidelines published by MHLW (Japanese Government partly followed  by the Declaration of Helsinki |
| 7. Czech Republic | 7. Namibia: Good clinical practice guidelines |
| 8. Germany | 8. Senegal: Law on code of ethic for health research- good clinical trial ICH. |
| 9. Ghana | 9. Turkey: ICH-GCP A |
| 10. Hungary | 10. USA: The Declaration of Helsinki is relevant but is not specifically relied upon. We  have no specific reference to DofH in any regulations. ICH E-6, which is an official FDA guidance, refers to the “principles” enunciated in the DoH, but not to any existing version. We have various rules on informed consent, monitoring safety, and Good Clinical Practices (ICH E-6) |
| 11. Ireland | 11. Zimbabwe: Helsinki Declaration is the minimum guideline. We also use ICH  guidelines for investigational new drugs, WHO guidelines, CIOMS |
| 12. Israel |  |
| 13. Japan |  |
| 14. Kenya |  |
| 15. Latvia |  |
| 16. Malaysia |  |
| 17. Namibia |  |
| 18. Republic of Belarus |  |
| 19. Saudia Arabia |  |
| 20. Senegal |  |
| 21. Slovakia |  |
| 22. Taiwan |  |
| 23. Tanzania |  |
| 24. The EU |  |
| 25. The Netherlands |  |
| 26. Turkey |  |
| 27. Uganda |  |
| 28. United Arab Emirates |  |
| 29. United Kingdom |  |
| 30. Zimbabwe |  |

Seite 1

| Question 4: For the approval of a new pharmaceutical drug, do you require placebo controls or standard therapy for comparison, in situations where effective treatment is available? | | | | |
| --- | --- | --- | --- | --- |
| a) Always placebo control | b) Placebo control only if disease is not life threatening | c) Placebo control if the only burden on the patient is transient discomfort | d) Always standard therapy | e) Other |
|  | 1. Argentina | 1. Botswana | 1. Cuba | 1. Austria |
|  | 2. Armenia | *2.* Chile: Only if in the design, rescue medications and other measures are considered. | 2. Czech Republic | 2. Argentina |
|  | 3. Botswana | 3. Czech Republic | 3. Ghana | 3. Germany: If no proven intervention is available |
|  | 4. Czech Republic | 4. EMA | 4. Kenya | 4. Japan: For application to FDA placebo may be used under the restriction specified by the Declaration of Helsinki. |
|  | 5. Hungary | 5. Ireland | 5. Malaysia | 5. Saudi Arabia: Always standard therapy, except in case of compelling and scientifically sound methodological reasons where the use of placebo is necessary to determine the efficacy or safety of an intervention and the patients who receive placebo or no treatment will not be subject to any risk  of serious or irreversible harm. Extreme care must be taken to avoid abuse of this option |
|  | 6. Israel | 6. Israel | 6. Namibia | 6. Turkey: RCT with placebo is preferable but it depends. |
|  | 7. Republic of Belarus | 7. Latvia | 7. Senegal | 7. UAE: both required are required (with Placebo and best available treatment), however, we approve drugs that are approved by any of FDA, MHRA, EMA, TGA, and similar authorities in developed countries. |
|  | 8. Slovakia | 8. Taiwan | 8. Tanzania | 8. USA: We accept a variety of kinds of controls, which are listed in our regs at 314.126. The choice depends on what is scientifically and ethically appropriate. ICH E-10 lays out basic principles on how to choose the control group. It is particularly interested in comparative trials intended NOT to  show superiority but to rule our unacceptable inferiority (so-called non- inferiority, or NI, trials).They are used when a placebo control would be ethically unacceptable AND the NI study credible design, which would rarely be the case for a symptomatic treatment. They are are often (almost always) used for antibiotic trials of serious infections, many cancer situations, and cardiovascular trials where an existing treatment is known to prevent death, MI, stroke, etc and so must be given to patients. In addition to ICH E_10 we published a draft guidance on this.ICH E-10 also discusses historical controls. |
|  | 9. United Kingdom | 9. The Netherlands | 9. UAE |  |
|  |  | 10. Germany | 10. Uganda |  |
|  |  |  | 11. Zimbabwe |  |

| Question 5: The Declaration of Helsinki has been revised several times since 1964. Does your organization adhere to a specific version of the Declaration of Helsinki? | | | | |  |
| --- | --- | --- | --- | --- | --- |
| a) 29th WMA General Assembly, Tokyo, Japan, October 1975 | b) 48th WMA General Assembly, Somerset West, South Africa, October 1996 | c) 52nd WMA General Assembly, Edinburgh, Scotland, October 2000 | d) 53rd WMA General Assembly, Washington DC, USA, October 2002 | e) 59th WMA General Assembly, Seoul, Korea, October 2008 | f) Other |
| 1. Senegal | 1. Czech Republic | 1. Namibia | 1. Tanzania | 1. Armenia | 1. Austria: No |
| 2. Tanzania | 2. Senegal | 2. Senegal | 2. Uganda | 2. Botswana (“We try to  use the latest version”) | 2. Argentina: Discussions are adhering  to the 59th. |
|  | 3. Tanzania | 3. Tanzania | 3. Senegal | 3. Chile | 3. Germany: 64th WMA General  Assembly, Fortaleza, Brazil, October  2013. Please note: In Article 3 of the  European Commission Directive  2005/28/EC the 1996 version of the  Declaration is referred to. |
|  | 4. Germany | 4. Zimbabwe |  | 4. Cuba | 4.Japan: Latest version |
|  |  |  |  | 5. EMA | 5. USA: The DoH was reasonably ok on the matter pf placebos until the  2000 version essentially banned placebo-controls when there was any existing therapy, even when no harm could come to the patient from use of placebo, an absurd position that showed complete ignorance of the problems of NI trials. This was largely  repaired in the 2008 version. Dr. Susan Ellenberg and I wrote about this in 200 (Annal Int Med) and the ICH E-10 guidance an NI guidance address  these matters. As noted, we no longer refer to the Declaration in any regulation. A placebo can always be used in an informed patient when no harm will come to the patient. It cannot be used when failure to use effective therapy could harm the patient. |
|  |  |  |  | 6. Ghana |  |
|  |  |  |  | 7. Hungary |  |
|  |  |  |  | 8. Ireland |  |
|  |  |  |  | 9. Israel |  |
|  |  |  |  | 10. Latvia |  |
|  |  |  |  | 11. Malaysia |  |
|  |  |  |  | 12. Republic of Belarus |  |
|  |  |  |  | 13. Saudi Arabia |  |
|  |  |  |  | 14. Senegal |  |
|  |  |  |  | 15. Slovakia |  |
|  |  |  |  | 16. The Netherlands |  |
|  |  |  |  | 17. Turkey |  |

| Question 6: How do you interpret paragraph 32 of the Declaration of Helsinki? | | |  |
| --- | --- | --- | --- |
| a) The use of placebo controls is appropriate in any circumstance. | b) The use of placebo control should be avoided whenever possible if effective treatment is available | c) The use of placebo is appropriate even if effective treatment exists but is not available in the location where the study is conducted. | d) Other |
|  | 1. Argentina | 1. Israel | 1. Austria: Ethical aspects of clinical trial submissions (interpretation of ethical principles, ethical justification of placebo) are within the remit of the Austrian Ethics Committees |
|  | 2. Armenia | 2. Senegal | 2. Cuba: Patients should never be randomized to treatment known to be inferior unless there are certain conditions that justify the use of the placebo (not necessarily unethical when proven effective treatment exists). |
|  | 3. Botswana | 3. Uganda | 3. Germany: As defined in the second  bullet point of paragraph 32: “Where for compelling and scientifically sound methodological reasons the use of placebo is necessary to determine the efficacy or safety of an intervention and the patients who receive placebo or no treatment will not be subject to any risk of serious or irreversible harm.” The agency takes care that when placebo treatment  is needed for methodological reasons the duration of the placebo treatment is as short as necessary. |
|  | 4. Chile | 4. United Kingdom | 4. Japan: Some people may  unfortunately consider the use of placebo is appropriate even if effective treatment is available. |
|  | 5. Czech Republic |  | 5.Saudi Arabia |
|  | 6. EMA |  | 6. Turkey: it depends disease and  existing treatment. |
|  | 7. Ghana |  | 7. USA: A=No, it is not appropriate if denial of available Rx causes harm.B= Not so.The study must be interpretable and in most symptomatic conditions, you need a placebo to interpret results (unless you are superior to an active control but you cannot count on that).C= Again, if the only consequence of not using standard therapy is discomfort we would consider the trial ethical in fully informed, non-coerced patients. Where there is treatment that affects outcome this is a difficult question and there is no specific FDA position. If the standard treatment is truly unavailable and cannot be administered in that location(e.g., a parenteral drug that can’t be administered, a surgical procedure they can’t manage), a placebo controlled trial of a treatment that could be used in that environment could be of value to the  location and would be considered ethical. |
|  | 8. Hungary |  |  |
|  | 9. Ireland |  |  |
|  | 10. Israel |  |  |
|  | 11. Kenya |  |  |
|  | 12. Latvia |  |  |
|  | 13. Malaysia |  |  |
|  | 14. Namibia |  |  |
|  | 15. Republic of Belarus |  |  |
|  | 16. Saudi Arabia |  |  |
|  | 17. Senegal |  |  |
|  | 18. Slovakia |  |  |
|  | 19. Taiwan |  |  |
|  | 20. Tanzania |  |  |
|  | 21. The Netherlands |  |  |
|  | 22. UAE |  |  |
|  | 23. Zimbabwe |  |  |

| Question 7: What are “compelling and scientifically sound methodological reasons” as outlined in paragraph 32 of the Declaration of Helsinki (see above) for your institution which would justify the use of placebo? | | |
| --- | --- | --- |
| Country | Answer to question |  |
| 1. Austria | See question 6 |  |
| 2. Argentina | It is an argument against each protocol, there are in general contrary opinions ... |  |
| 3. Armenia | Standard treatment is not very effective |  |
| 4. Botswana | This is when the available treatment is too expensive and can not be availes and the new candidate has potential to provide an answer or affordable alternative. Also a placebo can be considered if the condition being studied does not pose a serious threat to the trial participants if they end up on the place arm. |  |
| 5. Chile | Among others when the drug has a placebo effect, e.g.: antidepressants and if it is a short period of treatment and the subject is regularly monitored |  |
| 6. Cuba | In the evaluation of new treatments in psychiatric and in some neurological conditions with high subjective component. If there is an uncertainty about whether the available treatment is better than placebo. High rates of placebo response. Scientific merit and clinical value (early phase of efficacy assessment).Use of good informed consents and careful subjects monitoring plans |  |
| 7. Czech  Republic | preclinical data and previously clinical data |  |
| 8. EMA | The position of the Agency in relation to the use of placebo is reflected in: |  |
|  | A) Reflection paper on ethical and GCP aspects of clinical trials of medicinal products for human use conducted outside of the EU/EEA and submitted in marketing authorization applications to the EU Regulatory Authorities [http://www.ema.europa.eu/docs/en_GB/document_library/Regulatory_and_procedural_guideline/2010/06/WC500091530.pd](http://www.ema.europa.eu/docs/en_GB/document_library/Regulatory_and_procedural_guideline/2010/06/WC500091530.pdf)f |  |
|  | The use of placebo is permissible in accordance with principles foreseen in the Directive 2001/20/EC, Directive 2005/28/EC, the WHO (CIOMS) Guidelines 8 and 11, paragraph 32 of the Declaration of Helsinki (2008), article 23 of the Additional Protocol on Biomedical Research of the Council of Europe(2005), paragraph 2.1; 2.2; 2.3 and 2.12 of the Note for Guidance on Good Clinical Practice (CPMP/ICH/135/95), paragraphs 9.2.1 and 9.2.3 of the guideline on ethical considerations for clinical trials on medicinal products conducted with the pediatric population (2008) and ICH E10 (Choice of Control Group). The CPMP position statement on the use of placebo in clinical trials (28 June 2001 EMEA/17424/01) should also be taken into account. |  |
|  | B) CPMP position statement on the use of placebo in clinical trials (28 June 2001 EMEA/17424/01). [http://www.ema.europa.eu/docs/en_GB/document_library/Position_statement/2009/12/WC500017646.pd](http://www.ema.europa.eu/docs/en_GB/document_library/Position_statement/2009/12/WC500017646.pdf)f |  |
|  | in which the Agency expressed its view: placebo-controlled trials are considered essential to demonstrate the efficacy of some new medicinal products. |  |
|  | “Although the efficacy of some new medicinal products can be satisfactorily demonstrated without the use of a placebo, for others the judicious use of placebo remains essential to  demonstrate their value. Where medicinal products do exist for a given indication, active controlled trials are encouraged provided that a methodologically acceptable  demonstration of efficacy and safety can be obtained. However, trials that seek to prove that a new agent and an active control have similar efficacy are inherently less reliable than trials that seek to prove the superiority of the new agent to a comparator, whether inactive or active. Increasing the size of trials does not alleviate this problem. In some areas of medicine this lack of reliability means that it is only possible to obtain convincing scientific evidence of the efficacy of a new medicinal product by means of superiority trials. The  use of an active control in such an area of medicine would mean that a new product would always have to demonstrate an improvement in efficacy over a currently authorized treatment. This may be too restrictive as, for example, granting an authorization to a new medicinal product with similar efficacy and improved safety, may also be in the best interest of patients. |  |
|  | There are a number of conditions that govern and restrict the use of placebo in order to avoid un-ethical use. First and foremost, the period during which a placebo is administered must not entail any additional risk of irreversible harm to the patient. Also, the patient included in the trial, or his/her legal representative, must receive and understand appropriate information on the trial, and give informed written consent. The patient’s right to withdraw at any time, but still receive conventional treatment must be respected. It is acknowledged that un-ethical abuses of placebo in trials of medicinal products may occur in any country, and this potential for abuse should be eliminated. Similar ethical standards should be applied in trials performed in the European Union as well as in foreign countries. These aspects fall within the responsibilities of Ethics Committees reviewing protocols of clinical trials; they are also emphasized in ICH E6 guideline on Good Clinical Practice and in the Council Directive 2001/20/EC on Good Clinical Practice. |  |
|  | Forbidding placebo-controlled trials in therapeutic areas where there are proven prophylactic, diagnostic or therapeutic methods would preclude obtaining reliable scientific evidence for the evaluation of new medicinal products, and be contrary to public health interest as there is a need for both new products and alternatives to existing medicinal products. Reliable scientific evidence of efficacy and safety ensures that a reliable evaluation of the balance of benefits and risks for a particular medicinal product can be made, avoiding erroneous decisions of either withholding or mistakenly granting a marketing authorization. Provided that the conditions that ensure the ethical nature of placebo-controlled trials are clearly understood and implemented, it is the position of the CPMP and the EMEA that continued availability of placebo-controlled trials is necessary to satisfy public health needs. |  |
|  | C) NOTE FOR GUIDANCE ON CHOICE OF CONTROL GROUP IN CLINICAL TRIALS (CPMP/ICH/364/96[)http://www.ema.europa.eu/docs/en_GB/document_library/Scientific_guideline/2009/09/WC500002925.pd](http://www.ema.europa.eu/docs/en_GB/document_library/Scientific_guideline/2009/09/WC500002925.pdf)f |  |
| 9. Germany | This is generally a case-by-case decision whether the BfArM accepts the use of placebo or “no treatment” in clinical trials. One of the most frequent reasons for the use of placebo is to test the assay sensitivity in clinical test situations where the situation itself may substantially influence the results. This is done to avoid false positive results leading to falsely proven interventions (strong placebo effects anticipated). |  |
| 10. Ghana | When standard therapy has toxicity so severe that many patients have refused to receive it. When withholding standard treatment will not results in serious or irreversible harm to subject. When it is a common practice not to initiate drug therapy immediately after diagnosis, then the placebo could be used in that short window period before the drug therapy. When the use of a placebo is the most rigorous test of treatment efficacy for evaluating a medical therapy. When a placebo group can be used as an “add on” to standard of care in comparison to an investigational treatment added to standard of care. In all above, there should be a rescue treatment to the situation in the protocol. |  |
| 11. Hungary | We assess clinical studies according to scientific guidelines of EMA. |  |
| 12. Ireland | Please note that most frequently placebo is used in patients who are also receiving standard of care. So usually there are two arms - standard of care + new active treatment and standard of care + placebo. |  |
| 13. Israel | 1. The patient is not responding to the existing therapy, or does not tolerate existing therapy. |  |
|  | 2. The current treatment does not rely on sound scientific grounds |  |
|  | 3. Stopping existing therapy for the trial timetable is not going to cause harm to the patient. |  |
| 14. Japan | Though I regret to say that FDA may think appropriate statistical methods are not available to verify the efficacy of a new drug in case active control however I believe there are no justification of the use of placebo even from the statistical viewpoints. |  |
| 15. Latvia | If comparator (active treatment)represents e.g. pharmaco therapeutical group other than investigational medicinal product (IMP) - in this case placebo could be more appropriate to demonstrate the efficacy/safety of the IMP |  |
| 16. Malaysia | Use of placebo will depend on the aim(s) and objective(s) of the study. |  |
| 17. Namibia | No current proven intervention exists or the current intervention has been ineffective |  |
| 18. Republic of  Belarus | When there are no methods of proven effectiveness, or when withdrawal or withholding of such methods does not present an unacceptable risk or burden. While |  |
|  | there is no additional burden as a result of the participants’ condition on their families/careers; |  |
|  | the study doesn’t involve participants not able to consent; |  |
|  | there are measures in place for early detection of a seriously unfavorable course of the disease in participants on placebo that would necessitate appropriate intervention; |  |
|  | there is provision for an appropriate timely interim analysis after 1/3, 1/2 and 3/4 of patients have been included. |  |
| 19. Saudi Arabia | Where the trial methodology has minimal risks to subjects, for example: |  |
|  | · Exclude subjects with an increased risk of harm from non-response. |  |
|  | · Include in the protocol increased monitoring for subject deterioration and the use of rescue medications. |  |
|  | · "Early escape" mechanisms and explicit withdrawal criteria may be built in so subjects will not undergo prolonged placebo treatment if they are not doing well. |  |
|  | · The size of the population placed on placebo may be smaller than the number in active treatment arms. |  |
|  | · Placebo and active treatment may be compared in an "add-on" method, keeping the subjects on identical maintenance treatments and then adding on the active treatment to one arm and placebo to the other. This design is especially applicable when the available treatment is known to decrease mortality or morbidity. |  |
|  | · Shortened treatment periods reduce the risks associated with delayed treatment. In situations in which long-term placebo treatment would not be acceptable, the use of a placebo group for a short period at the beginning of a trial could establish short-term effects. The trial would then continue without the placebo group. |  |
|  | · Unblinded data review by a Data Safety Monitoring Board with interim analysis of study results and safety issues. This is especially important for multi-center site studies. |  |
| 20. Senegal | The use of placebo in clinical trials necessary to obtain a control group with an analysis of single-blind (patient does not know what it receives) or double-blind (neither the doctor nor  the patient know what is given. the patient should not be in danger(life threat) |  |
| 21. Slovakia | It depends on Design, phase of IMP development and aim of the study. |  |
| 22. Taiwan | No comment, for we do not entirely follow the current version of the Declaration of Helsinki of 2008. |  |
| 23. Tanzania | It will depend on the intervention itself and the design eg if there is evidence from the pharmacovigilance data or studies that proves that the standard treatment is too harmful or  toxic or not tolerable |  |
| 24. The  Netherlands | Scientifically, a direct comparison between treatment and placebo is the most informative design. However, an MD should always act in the best interest of the patient which indicates that also a control group should have access to standard treatment. The use of placebo may be acceptable when a placebo-effect should be quantitated in relation to any specific effect of an intervention. For the subject/patient however, this is not relevant and subsequent research may be needed to compare the new and the standard treatment anyhow. |  |
| 25. UAE | Standard or best available comparator |  |
| 21. Uganda | Where no effective treatment exists or where the treatment is too expensive or not available in Uganda. |  |
| 22. United Kingdom | These vary |  |
| 23. USA | If an NI study is not interpretable because there is no valid grounds for choosing an NI margin, the NI design cannot be used (and an invalid design is unethical) and a placebo  control is ethical if the patient will not be harmed (see ICH E-10) and is fully informed and not coerced. As a practical matter, almost all symptomatic treatments are studied in a placebo-controlled or dose-response studies, i.e., studies intended to show a difference between treatments. |  |
| 24. Zimbabwe | Not applicable, we only approve use of placebo when no proven intervention exists. |  |

| Question 8: How does your institution define “serious harm” as outlined in paragraph 32 of the Declaration of Helsinki (see above) | |
| --- | --- |
| for a patient which would restrict the use of placebo? | |
| Country | Answer to question |
| 1. Austria | See question 6 |
| 2. Argentina | Each protocol must be evaluated by an independent ethics committee, and it is their responsibility to justify the use  of placebo or not. |
| 3. Armenia | Strengthening of the disease if left untreated. |
| 4. Botswana | Serious harm includes worsening of medical condition due to non-treatment, complications including hospitalization. |
| 5. Chile | The definition is consistent with the one of the WHO |
| 6. Cuba | Serious adverse events or unexpected. More risks than benefits |
| 7. Czech Republic | Using a placebo must not be delayed basic treatment and must not endanger the health of the patient, each study  is assessed separately(case by case) |
| 8. EMA | Serious harm can be considered events resulting in death or life-threatening, requires inpatient hospitalization or  results in prolongation of existing hospitalization, results in persistent or significant disability/incapacity, results in a medically important event or reaction. Medical and scientific judgment should be exercised in deciding whether other situations should be considered serious such as important medical events that might not be immediately life- threatening or result in death or hospitalization but might jeopardize the patient or might require intervention to prevent one of the other outcomes listed above*.* |
| 9. Germany | This is also a case-by-case decision which takes several other considerations into account, such as the underlying  disease, the available medical options as well as the patient situation itself. Usually, the following conditions would  be considered as “serious harm”. If the harm could |
|  | - be life-threatening |
|  | - lead to irreversible permanent damages or disability |
|  | - lead to a hospitalization or prolongation of a hospitalization |
|  | - impact the fertility or may damage unborn life or could cause birth defects |
|  | - be likely to cause an important medical event (as defined in ICH E2A guideline) |
| 10. Ghana | We have no definition for “serious harm”, however, when there is any undesirable experience associated with the use of a medical product in a patient, where it results in death, life threatening, hospitalization, disability or permanent damage or congenital anomaly, it is termed as “Serious Adverse Event” as per our local guidelines. |
| 11. Hungary | The life threatening situations or maintenance therapy of schizophrenic patients. |
| 12. Ireland | As stated above - the use of placebo control should be avoided whenever possible if effective treatment is  available. |
| 13. Israel | A change in his/hers medical condition that requires a change in his/hers medical care. |
| 14. Japan | Significant and irreversible risk to the patients |
| 15. Latvia | "Serious harm"-permanent worsening of the patient's condition or clinically significant deterioration. Serious harm not to be allowed. |
| 16. Malaysia | Subject(s) is/are denied treatment for their condition which may worsen over the course of the study. |
| 17. Namibia | Results in death, is life threatening. Requires patient hospitalization or prolongation of hospitalization. Results in persistent or significant disability/incapacity or congenital anomaly / birth defect |
| 18. Republic of  Belarus | It should be taken into account: |
|  | absence of mortality from the disease itself and its complications without pharmacotherapy during the  observation period and the long-term period (12 months); |
|  | absence of the vital target organ damage without pharmacotherapy in the acute phase of trials; |
|  | absence of a temporary or permanent disability during the period without treatment and long-term period (12  months). |
| 19. Saudi Arabia | In case of: |
|  | 1. Death. |
|  | 2. Life-threatening. |
|  | 3. Requires inpatient hospitalization or causes prolongation of existing hospitalization. |
|  | 4. Results in persistent or significant disability/incapacity. |
|  | 5. Congenital anomaly/birth defect, or |
|  | 6. Requires intervention to prevent permanent impairment or damage. |
| 20. Senegal | No official definition |
| 21. Slovakia | Risk of progressions or worsening of the disease. |
| 22. Taiwan | No comment, for we do not entirely follow the current version of the Declaration of Helsinki of 2008. |
| 23. Tanzania | Serious harm such as death or irreversible morbidity |
| 24. The  Netherlands | When standard treatment is either live-saving or live-extending, and when any abstention of treatment is very  discomforting and/or results in grave and irreversible damage. |
| 25. UAE | Clinical consequence that needs hospitalization |
| 26. Uganda | Serious harm that would cause disability, is life threatening, causes or prolongs hospitalization. |
| 27. United  Kingdom | There is no standard definition of harm. A decision would normally be taken on a case by case basis |
| 28. USA | We generally mean death or irreversible morbidity. We recognize that there are levels of discomfort that would trouble people and they might decide this was unacceptable. Of course, you can introduce early escape provisions. If a placebo control cannot be used that does not on any way suggest that an active control would be informative. You may just not be able to develop a new drug unless it was actually superior. |
| 29. Zimbabwe | Not applicable. |


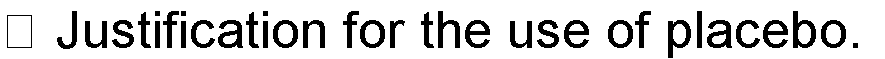

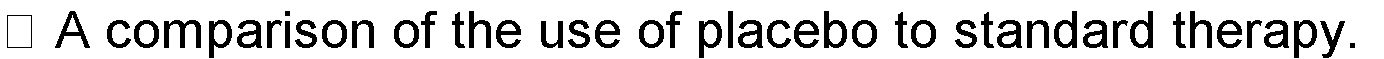

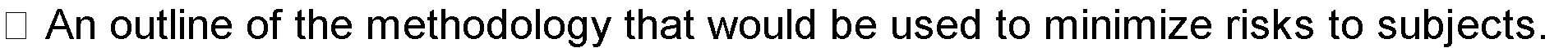


| Question 9:Which measures does your institution take “to avoid abuse of this option” as outlined in paragraph 32 of the Declaration of Helsinki (see above)? | |
| --- | --- |
| Country | Answer to question |
| 1. Austria | When a clinical trial does not receive a positive Ethics Committee opinion, the trial is not authorized by BASG/AGES and can therefore not be initiated in Austria. |
| 2. Argentina | Before conducting the clinical study, the protocol must be approved by the ethics committee and it is them who decide on the  subject. If ANMAT doesn’t agree, the clinical study is not allowed in the country. |
| 3. Armenia | We do our best for avoiding of placebo in case of availability standard treatment |
| 4. Botswana | Exploration of alternative ways including possibility of providing the proven treatment even when that treatment is not available in  Botswana. |
| 5. Chile | Must be very limited to: - when there is no standard treatment known to be effective or when required by scientists. Precautions have to be taken: short period of time, rescue medication, close monitoring and other measures |
| 6. Cuba | The use of a committee of quality (with high qualified specialists)to evaluate the proposed designs of all the clinical trials we perform, ethics committees in all the clinical sites with well-trained members to review each clinical trial protocol, to analyze from the statistical and methodology point of views if the use of placebo is justified |
| 7. Czech Republic | We require to submit substantial amendment with change to protocol (design, visit, exclusion and inclusion criteria or withdraw placebo). If it is not possible or sponsor does not want to change documentation, we will reject the clinical trial application |
| 8. EMA | EMA in the Reflection Paper clarified the following : |
|  | - Studies carried out in countries outside EU/EEA should meet the same ethical principles and standards applied to studies performed in the EEA. Derogation from these principles should not be accepted in particular in the context of the European marketing authorization procedure. |
|  | - EU Regulatory Authorities should neither require nor accept study designs, involving placebo or other comparator, which would not be ethically acceptable in the EEA. |
|  | - Economic [or logistical] reason for the unavailability of an established effective intervention cannot justify a placebo-controlled study in a country of limited resources when it would be unethical to conduct a study with the same design in a population with general access to the effective intervention outside the study”. |
|  | - Lack of access of patients in communities within, or outside of, the EEA, to the EEA-licensed (or equivalent) comparator cannot be a justification to withhold this treatment option to those patients when participating in a trial regardless of the reasons for the lack of access (e.g. no reimbursement, no national marketing authorization). Regardless of the location of the trial, all patients participating in these trials should receive the same or a similar standard of care and comparable treatment options as trial participants within the EEA. |
|  | - EU Regulatory Authorities should verify that the study has been reviewed by the Ethics Committees and that they have determined: whether the use of placebo or other comparator is ethically acceptable in the context of that trial; whether the safety and rights of the subjects have been fully protected and whether prospective subjects would be fully informed about the use of placebo and/or other comparators and available alternative treatments and gave their informed consent (or informed refusal), in accordance with above cited ethical principles. |
|  | EMA/ EU Competent Authorities actions taken to avoid abuse of placebo include : |
|  | - Sponsors should describe in detail in the protocol and in the clinical study report the justification for the use of placebo and/or choice of active comparator in accordance with the ethical principles referred to above. This information can form part of the clinical study report in accordance with ICH3 and protocol in accordance with ICH E6. |
|  | - EU Regulatory Authorities will identify those studies that may give rise to special ethical concern regarding the use of placebo or other comparators and where applicable seek additional assurance that the design was appropriate and ethically acceptable. |
|  | - Where it is determined that a study design was not acceptable in accordance with the aforementioned criteria, it should not be accepted in support of a MAA in accordance with Directive 2001/83 EC and Regulation No (EC) 726/2004. |
|  | - If a sponsor has particular concern about a particular trial design, it is strongly advised to seek scientific advice with Regulators on study design before carrying out the trials. |
| 9. Germany | Each trial protocol is intensively reviewed and the agency pays attention that the use of placebo is restricted to a minimum of trials according to paragraph 32 of the Declaration. In most cases the use of placebo is performed as add-on to a standard therapy, i.e. all trial subjects receive the standard treatment and additionally a new compound or placebo on top. |
| 10. Ghana | It is a requirement to include the following information in the protocol: |
|  |  |
|  |  |
|  |  |
| 11. Hungary | The usage of placebo is a very important aspect in our assessment |
| 12. Ireland | Clinical trials are assessed independently by ethics committees in Ireland. |
| 13. Israel | We ask for a letter of explanation on the need to have a placebo group. |
| 14. Japan | Our institute basically will not support the use of placebo when the active control is available. |
| 15. Latvia | Strong and careful assessment of risk/benefit for the patient (in case by case manner) |
| 16. Malaysia | We will review both the scientific and ethical aspects of the study. |
| 17. Namibia | The Biomedical Research Ethics Committee (BREC) conducts initial and continuing review of all clinical research activities in  Namibia to detect any serious non-compliance with the regulations |
| 18. Republic of  Belarus | Expertise and approval of documents for clinical trials by regulatory authorities (Ministry of Health, Republican Unitary Enterprise «Center for Examinations and Tests in Health Service»), subject matter specialists. Ethical expertise and approval of documents for clinical trials by local Ethics Committee of trial sites. |
| 19. Senegal | There is a review of all studies conducted in the country by an ethical and scientifically committee before any authorization. |
| 20. Slovakia | The use of placebo is always subject of our assessment of any clinical trial application. In some cases we ask for more detail explanation of the need of placebo. |
| 21. Taiwan | No comment, for we do not entirely follow the current version of the Declaration of Helsinki of 2008. |
| 22. Tanzania | By careful assessment of the data and evidence before clinical trial authorization and by conducting inspections after approval to ensure that participants are not at risk. |
| 23. The Netherlands | Any research proposal should be accompanied by all relevant information on existing (effective) treatments and broadly accepted  as yet unproven effective treatments. Next, a comprehensive justification should be provided on the acceptability of the placebo- arm for methodological or pragmatic reasons when an effective treatment is available. This will be part of the overall ethical and medical assessment and be a crucial aspect in the final outcome thereof. |
| 24. UAE | Best available evidence/ systemic review |
| 25. Uganda | Various levels of review. Peer review by scientific and ethics review board, review and registration by Uganda national council for science and technology. Review by NDA. |
| 26. United Kingdom | None |
| 27. USA | We don’t think it is abused but we see all trial proposals before they are implemented. We would not allow a trial that could lead to  irreversible morbidity or death from denying standard treatment. |
| 28. Zimbabwe | Not applicable. |

| Question 10: Do you have any additional comments on any of our questions above? | |
| --- | --- |
| County | Answer to question |
| 1. Austria | No |
| 2. Argentina | This is a very controversial issue among methodologists and ethicists. |
| 3. Botswana | Each case has to be considered for its merits as there may be other factors that can influence the decision. |
| 4. Chile | We have no specific guidelines in our country so we rely primarily to the guidelines of the EMA |
| 5. Czech Republic | Ethics Committees assess ethical aspect in the clinical trials in the Czech Republic. |
| 6. EMA | Please refer to the following three documents for further details: |
|  | [- Reflection paper on ethical and GCP aspects of clinical trials of medicinal products for human use conducted outside of the](http://www.ema.europa.eu/docs/en_GB/document_library/Regulatory_and_procedural_guideline/2010/06/WC500091530.pdf)  [EU/EEA and submitted in marketing authorization applications to the EU Regulatory Authorities](http://www.ema.europa.eu/docs/en_GB/document_library/Regulatory_and_procedural_guideline/2010/06/WC500091530.pdf) [http://www.ema.europa.eu/docs/en_GB/document_library/Regulatory_and_procedural_guideline/2010/06/WC50009](http://www.ema.europa.eu/docs/en_GB/document_library/Regulatory_and_procedural_guideline/2010/06/WC500091530.pdf)1530. pdf |
|  | [- CPMP position statement on the use of placebo in clinical trials (28 June 2001 EMEA/17424/01).](http://www.ema.europa.eu/docs/en_GB/document_library/Position_statement/2009/12/WC500017646.pdf)  [http://www.ema.europa.eu/docs/en_GB/document_library/Position_statement/2009/12/WC500017646.pd](http://www.ema.europa.eu/docs/en_GB/document_library/Position_statement/2009/12/WC500017646.pdf)f |
|  | - NOTE FOR GUIDANCE ON CHOICE OF CONTROL GROUP IN CLINICAL TRIALS (CPMP/ICH/364/96) |
|  | [http://www.ema.europa.eu/docs/en_GB/document_library/Scientific_guideline/2009/09/WC500002925.pd](http://www.ema.europa.eu/docs/en_GB/document_library/Scientific_guideline/2009/09/WC500002925.pdf)f |
|  |  |
| 7. Ghana | None |
| 8. Ireland | Please note that an ethical consideration of a clinical trial is assessed independently by ethics committees in  Ireland. |
| 9. Israel | In a situation of global trials a small country like Israel is going in line with what was drawn by the FDA or EMA |
| 10. Latvia | No |
| 11. Malaysia | No |
| 12. Republic of Belarus | No comments |
| 13. Saudi Arabia | No |
| 14. Taiwan | No comment, for we do not entirely follow the current version of the Declaration of Helsinki of 2008. |
| 15. The Netherlands | No |
| 16. UAE | Good luck |
| 17. Uganda | No |
| 18. USA | No |

Response of Health Canada

Please be advised that Health Canada has not adopted the Declaration of Helsinki. Rather,we refer clinical trial sponsors to the Regulations that govern clinical research (Drugs For Clinical Trials Involving Human Subjects), as well as the internationally-recognized guidelines developed by the International Conference on Harmonisation (ICH) - Good Clinical Practices E6(R1) (May 1996). ICH E6 is the accepted standard for the design, conduct, recording and reporting of drug clinical trials that

involve the participation of human subjects.

With respect to the ethics of placebo control, it is never as simple as where one draws the line. When one considers the ethics of placebo control, one should also consider the ethics of the alternatives of placebo control. When the conditions are not favourable to the successful trial completion using an active control, such a trial is of questionable ethics since it cannot return useful information and exposes patients to unnecessary risks. Scientific consideration is an essential part of

ethical deliberation.

When proven effective therapy is available, Health Canada considers placebo control as permissible although not always necessary, when the withholding

of proven effective therapy is not expected to expose patients to risks of serious or irreversible harm. It should be pointed out that the use of an unproven active test therapy is withholding proven effective therapy, unless it is an add-on design. After it has been determined that placebo control is permissible, its choice, as with the choice of active control,

is to be justified scientifically.

The need for placebo control depends on a number of factors. The main factors include therapeutic area, indication sought, expected placebo response rate, anticipated therapeutic effect size, superiority or

non-inferiority trial objective, availability of proven effective therapy, performance of chosen active control, and risk of withholding proven effective therapy.

Conditions that are favourable to the successful use of active control include: low placebo response; effects of active control high, stable, and predictable; test therapy is expected to be similar to or better than

active control. Either superiority or non-inferiority trials may be conducted. Placebo control is not necessary.

Conditions that are not favourable to the successful use of active control include: high placebo response; effect of active control low to moderate, variable, and not very predictable; test therapy is expected to be similar to active control. In these cases, placebo control is necessary and

non-inferiority trials should not be attempted.

Please refer to Figure 1 of ICH E-10 for options for choice of control based on both scientific and ethical considerations. Use of both active and placebo controls in a 3-arm trial can be considered when ethically permissible. ICH E10 and covering Health Canada notice are available at the following link:

[http://www.hc-sc.gc.ca/dhp-mps/prodpharma/applic-demande/guide-ld/ich/efficac/e10_step4-eng.ph](http://www.hc-sc.gc.ca/dhp-mps/prodpharma/applic-demande/guide-ld/ich/efficac/e10_step4-eng.php)p
